# Impact of Lipoprotein(a) on Residual Cardiovascular Risk After an Acute Coronary Syndrome

**DOI:** 10.3390/jcm15051688

**Published:** 2026-02-24

**Authors:** Nelsa González-Aguado, Rafael Franco-Hita, Jose Ignacio Larrubia-Valle, Fernando Puyol-Ruiz, Ainhoa Robles-Mezcua, José Manuel García-Pinilla, María Jiménez-Salva, Alberto Piserra-López, Francisco Javier Pavon-Moron, Alejandro Pérez-Cabeza, Pierre Sabouret, Francesco Costa

**Affiliations:** 1 Department of Medicine, UMA (Universidad de Málaga), Heart Area, Hospital Universitario Virgen de la Victoria, CIBERCV (Centro de Investigación Biomédica en Red Enfermedades Cardiovaculares), IBIMA Plataforma BIONAND (Instituto de Investigación Biomédica de Málaga y Plataforma en Nanomedicina), 29010 Malaga, Spain; 2 Centro de Investigación Biomédica en Red de Enfermedades Cardiovasculares (CIBERCV), 28029 Madrid, Spain; 3 Heart Institute, ACTION Group, Pitié-Salpétrière Hospital, Sorbonne University, 47–83 Boulevard de l’Hôpital, 75013 Paris, France; 4 Department of Biomedical and Dental Sciences and of Morphological and Functional Images, University of Messina, 98122 Messina, Italy

**Keywords:** lipoprotein(a), acute coronary syndrome, residual cardiovascular risk, major cardiovascular events, emerging therapies

## Abstract

Reducing residual cardiovascular risk following acute coronary syndrome (ACS) remains a major unmet clinical need. Despite substantial advances in lipid-lowering therapies, the risk of recurrent major adverse cardiovascular events (MACEs) after ACS remains high, with an estimated incidence of approximately 33.4% at 5 years. Residual cardiovascular risk is driven by multiple mechanisms, including persistent inflammation, a prothrombotic status, metabolic disturbances, and the presence of atherogenic lipoproteins beyond low-density lipoprotein cholesterol (LDL-C). Lipoprotein(a) (Lp(a)) is a pro-inflammatory, prothrombotic, and pro-atherosclerotic lipoprotein that appears to play a major role in residual risk after ACS or ischemic stroke. Elevated Lp(a) is a well-established independent and causal risk factor for atherosclerotic cardiovascular disease (ASCVD). Nevertheless, evidence regarding its prognostic value specifically after ACS remains limited, with marked heterogeneity across studies, which complicates direct comparisons and interpretation. In addition, while Lp(a) levels are predominantly genetically determined, recent studies have reported intra-individual variability, although their clinical significance remains uncertain. Finally, current therapeutic options specifically targeting Lp(a) are limited. Novel RNA-based therapies, including antisense oligonucleotides, small interfering RNAs, and emerging gene-editing approaches, have demonstrated profound and sustained reductions in circulating Lp(a) levels. Yet, whether this biological effect translates into reductions in hard clinical endpoints is under evaluation in ongoing clinical trials. This review aims to synthesize current evidence on the role of Lp(a) as a major contributor to residual cardiovascular risk following ACS.

## 1. Introduction

Despite advances in treatment over the last few decades, the management of residual cardiovascular risk after acute coronary syndrome (ACS) is still a major challenge. Patients with ACS continue to face a substantial risk of recurrent major adverse cardiovascular events (MACEs), with an estimated event rate of approximately 33.4% at 5 years of follow-up, and a six-fold higher risk during the first year after diagnosis [1].

Traditionally, secondary prevention strategies have been focused on multiple risk pathways, especially on low-density lipoprotein cholesterol (LDL-C) reduction. Nevertheless, evidence from randomized controlled trials (RCTs), such as the *ODYSSEY Outcomes* trial, which evaluated the Proprotein Convertase Subtilisin/Kexin Type 9 (PCSK9) inhibitor alirocumab in patients following an ACS, has demonstrated that despite achieving intensive LDL-C control, residual risk of cardiovascular events remains substantial [2]. As a consequence, recent studies advocate the need for a more holistic approach to residual cardiovascular risk beyond traditional factors [3,4].

Lipoprotein(a) (Lp(a)) has emerged as an independent risk factor of atherosclerotic cardiovascular disease (ASCVD) and calcific aortic valvular disease (CAVD). Several mechanisms explain its pathogenic role, including proatherogenic, pro-inflammatory, and prothrombotic effects [5,6,7]. Therefore, the last European Society of Cardiology (ESC)/European Atherosclerosis Society (EAS) and American Heart Association (AHA) guidelines identify high Lp(a) levels (≥50 mg/dL) as a risk modifier and recommend measuring Lp(a) at least once during an individual’s lifetime [8,9].

Furthermore, growing evidence identifies Lp(a) as an important determinant of residual cardiovascular risk after an ACS. Elevated Lp(a) levels have been associated with premature and more severe ACS, with a higher risk of recurrent ischemic events after the index event, both early after the index event and during long-term follow-up [10]. Nonetheless, available data remain heterogeneous, with substantial variability in study design, patient populations, outcome definitions, and Lp(a) cut-off values, likely reflecting the linear association between Lp(a) levels and MACE rather than a clear risk threshold; consequently, robust long-term prospective evidence in ACS cohorts is still limited.

Importantly, therapeutic options specifically targeting Lp(a) are currently limited. Nevertheless, ongoing clinical trials evaluating antisense oligonucleotides (ASO) and small interfering RNA (siRNA) therapies have demonstrated substantial reductions in Lp(a) concentrations; whether these biomarker effects will translate into clinically meaningful cardiovascular benefit has yet to be determined [11].

This review aims to provide a comprehensive overview of Lp(a) as a key marker of residual cardiovascular risk in the specific context following ACS, highlighting its intra-individual variability and the emerging cardiovascular outcomes trials. 

## 2. Lipoprotein(a): Structure, Metabolism and Pathophysiological Mechanisms

Lp(a) is a plasma macromolecular complex, first described in 1963 [12,13], consisting of an LDL particle containing apolipoprotein B-100 (apoB-100) covalently linked by a single disulfide bridge to apolipoprotein(a) (apo(a)) [14].

**Figure 1:**
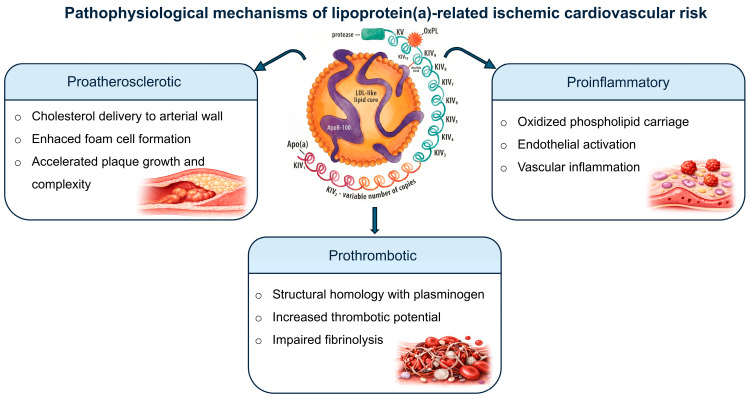
Pathophysiological Mechanisms of Lipoprotein(a). The central figure represents a lipoprotein(a) molecule with its detailed structure.

From a perspective, Lp(a) exhibits a triad of pro-atherogenic, pro-inflammatory, and pro-thrombogenic properties (Figure 1) [14,15,16,17,18,19]. It contributes to atherosclerosis via cholesterol deposition within the arterial intima and acts as a preferential carrier of oxidized phospholipids, which promote vascular inflammation, endothelial dysfunction, and plaque instability [16]. In addition, apo(a) exhibits marked structural homology with plasminogen and other coagulation factors, as it contains multiple loop-like structures known as “kringle domains”, competitively inhibiting plasminogen binding and activation, and promoting a prothrombotic state [17,18,19]. These synergistic effects contribute to the strong and causal association between elevated Lp(a) levels and ASCVD, including coronary artery disease (CAD), stroke, and CAVD [20,21,22].

## 3. Genetic Determinants and Variability of Lipoprotein(a)

Lp(a) concentration is predominantly genetically determined, with heritability estimated at 70–90%. The *LPA* gene on chromosome 6 is the principal genetic determinant and encodes apo(a).

Among apo(a) kringle domains, the KIV-2 copy number variation (CNV) accounts for a substantial proportion of interindividual variability, and it inversely correlates with Lp(a) concentration: fewer repeats result in smaller apo(a) isoforms, and higher Lp(a) levels. The underlying mechanism involves prolonged retention of large apo(a) isoforms in the endoplasmic reticulum, where they undergo increased proteasomal degradation, whereas smaller isoforms are more efficiently secreted.

Beyond CNVs, numerous single-nucleotide polymorphisms within the *LPA* locus further modulate Lp(a) levels, even among individuals with identical KIV-2 repeat numbers. Notably, plasma levels differ across racial and ethnic groups, with higher concentrations observed in African and South Asian populations. These differences are largely attributed to population-specific genetic variations affecting apo(a) isoform size and *LPA* gene structure [5,23,24].

### Intra-Individual Variability

Intra-individual variability of Lp(a) concentrations is generally low, with most individuals maintaining stable levels over time, especially those with clearly low (<30 mg/dL) or high (≥50 mg/dL) baseline values. Nonetheless, recent studies have reported significant intra-individual variability (>10 mg/dL or >25%), particularly in patients with borderline levels (30–50 mg/dL) [25,26,27,28,29,30,31,32,33,34,35,36].

As early as 1995, Nakajima et al. demonstrated an intra-individual variability of Lp(a) of 16.6%, identifying lipid metabolism-related factors, platelet-mediated prothrombotic pathways, and acute-phase reactants as key determinants of this variability [25]. In recent years, several studies have reported intra-individual Lp(a) changes exceeding 10 mg/dL or 25% in a substantial proportion of individuals, affecting approximately 15–40% of the population depending on the study. This variability may be clinically relevant for cardiovascular risk stratification, particularly within the “gray zone,” where up to 50% of individuals may be reclassified to higher or lower risk categories following repeat measurement. Nevertheless, whether intra-individual variability in Lp(a) is associated with adverse cardiovascular outcomes remains uncertain [26,27,28,29,30,31,32,33,34,35,36]. Recently, Trinder et al. found an association between follow-up Lp(a) levels and incident CAD, whereas intra-individual variability was not independently associated with risk beyond follow-up Lp(a) levels [26].

**Figure 2:**
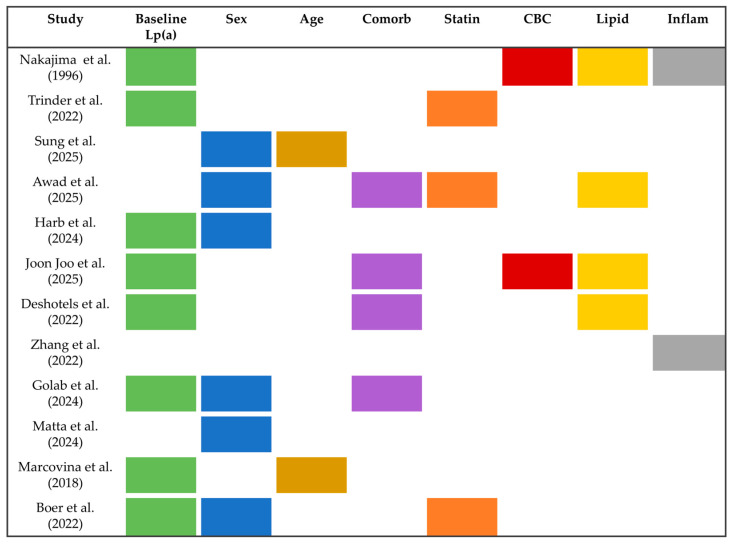
Comprehensive summary of clinical and laboratory predictors for lipoprotein(a) variability evaluated across studies [25,26,27,28,29,30,31,32,33,34,35,36]. Colored boxes indicate that the corresponding variable was evaluated in the study. Each color represents a predictor of variability to facilitate visualization and improve interpretability. Abbreviations: Lp(a): Lipoprotein(a); Comorb: Comorbidities (ASCVD, Hypertension, Diabetes, Smoking, BMI (Body Mass Index), SCORE2 (Systematic Coronary Risk Estimation 2)); CBC: Complete Blood Count (Platelets, Hemoglobin, White Blood Cells); Lipid: lipid profile (Total Cholesterol, LDL-C, HDL-C (High-Density Lipoprotein Cholesterol), Triglycerides, Apolipoproteins, Phospholipids); Inflam: Inflammatory Markers (Erythrocyte Sedimentation Rate, High-Sensitivity C-Reactive Protein (hsCRP)).

Several factors have been described as associated with increased Lp(a) variability; however, findings have not been consistent across all published studies (Figure 2). The factors with the strongest and most reproducible evidence include baseline Lp(a) concentration, female sex, advanced age, the presence of cardiovascular comorbidities, lipid profile, and concomitant statin use [25,26,27,28,29,30,31,32,33,34,35,36].

These findings support that a second measurement may be necessary in selected cases to optimize cardiovascular risk management, especially in patients with intermediate values or clinical factors associated with higher Lp(a) variability.

## 4. Association Between Elevated Lipoprotein(a) Levels and Atherosclerotic Cardiovascular Disease

Large contemporary cohort studies demonstrate a continuous and largely linear association between Lp(a) levels and ASCVD risk. In the UK Biobank analysis of nearly half a million participants, each 20 mg/dL increment in Lp(a) conferred an 11% increased ASCVD risk [hazard ratio (HR) 1.11, 95% confidence interval (CI) 1.10–1.12, per 20 mg/dL increment], with consistent relative risk across ethnic groups despite varying baseline Lp(a) levels [37]. Comparable dose–response relationships have been reported in recent meta-analyses of population-based and clinical cohorts [38,39].

For clinical decision-making, multiple consensus statements and cohort analyses converge on ≥50 mg/dL as a pragmatic high-risk threshold for clinical risk stratification, adopted by European and American guidelines as a risk-enhancing factor. Evidence shows that cardiovascular risk increases more sharply above this level, supporting its use as a risk-enhancing factor in clinical stratification. However, randomized cardiovascular outcome trials typically use a higher threshold, usually ≥70–80 mg/dL, to select populations at higher absolute risk, thereby maximizing statistical power and clinical relevance [8,9,38,39,40].

A main consideration is that Lp(a)-associated risk is independent of, and additive to, LDL-C-mediated risk. Even among statin-treated individuals with low achieved LDL-C, elevated Lp(a) continues to confer excess ASCVD risk, supporting an additive “double-hit” model [39]. Notably, methodological analyses indicate that adjusting LDL-C for the cholesterol content of Lp(a) does not meaningfully improve CAD risk prediction, underscoring the distinct prognostic role of Lp(a) [41]. Moreover, Björnson et al. suggested that, on a per-particle basis, the proatherogenic effect of Lp(a) may be substantially greater than that of LDL-C. Notably, they reported an approximately six-fold stronger association with atherosclerotic burden for Lp(a) compared with LDL-C [42].

Mortality data further support the prognostic relevance of Lp(a) beyond nonfatal ASCVD events [38,43]. A systematic review and dose–response meta-analysis demonstrated a largely linear association between Lp(a) levels and both cardiovascular and all-cause mortality in the general population and in individuals with established ASCVD [38].

These observations align with emerging evidence linking Lp(a) to “panvascular” atherosclerotic phenotypes, plausibly contributing to cause-specific mortality beyond coronary outcomes alone [44].

Moreover, elevated Lp(a) levels are associated with more severe, extensive, and premature coronary disease [45,46,47,48].

Moving from association to implementation, real-world data support the incorporation of Lp(a) into ASCVD risk prediction models. In a recently developed model of ASCVD risk in individuals without prior cardiovascular disease, higher Lp(a) levels were associated with progressively increased risk, with each 25 mg/dL increment conferring a 23% higher composite ASCVD risk (adjusted HR 1.23, 95% CI 1.10–1.37) [49]. Incorporation of Lp(a) improves model discrimination and results in meaningful reclassification of individuals at borderline or intermediate risk, highlighting potential implications for preventive interventions [49,50].

## 5. Lipoprotein(a) and Residual Cardiovascular Risk

Despite optimal control of traditional risk factors, RCTs evaluating PCSK9 inhibitors, such as the *ODYSSEY Outcomes* trial, have demonstrated that a substantial proportion of patients continue to experience recurrent cardiovascular events even under intensive LDL-C control [2], highlighting additional pathophysiological mechanisms involved in atherosclerotic progression. Residual cardiovascular risk is thus recognized as a multifactorial phenomenon driven by uncontrolled dyslipidemia, persistent inflammation, metabolic disturbances, and a prothrombotic state [3,4,51].

Beyond LDL-C, lipid-related residual cardiovascular risk includes triglycerides (TG), triglyceride-rich lipoproteins, and Lp(a), all of which contribute to atherosclerosis progression and adverse cardiovascular outcomes [52]. Over recent years, Lp(a) has emerged as a major contributor to residual cardiovascular risk across both acute and chronic cardiovascular disease [10,39,53,54,55,56,57,58,59,60,61,62,63,64,65,66,67,68,69,70,71,72,73,74].

### 5.1. Lipoprotein(a) as a Driver of Residual Cardiovascular Risk in Established Atherosclerotic Cardiovascular Disease

In patients with established ASCVD, the most comprehensive evidence comes from participant-level meta-analysis of statin trials. Willeit et al. analyzed 29,069 patients from seven placebo-controlled statin trials and demonstrated that elevated Lp(a) independently predicted cardiovascular events in statin-treated patients. Notably, as LDL-C–attributable risk was reduced, Lp(a)-associated risk became a stronger determinant of residual risk, particularly at concentrations exceeding 50 mg/dL. In this analysis, the HR for cardiovascular events was 1.11 (95% CI, 1.00–1.22) for Lp(a) levels of 30–50 mg/dL and increased to 1.58 (95% CI, 1.08–1.58) for concentrations ≥ 50 mg/dL. Correspondingly, among statin-treated patients, the HRs were 1.06 (95% CI, 0.94–1.21) for Lp(a) levels 30–50 mg/dL, and 1.43 for Lp(a) ≥ 50 mg/dL (95% CI, 1.15–1.76) [53].

These findings were recently confirmed by Bhatia et al. in a 2025 participant-level meta-analysis including 27,658 patients from six statin trials. Elevated Lp(a) (>50 mg/dL) was associated with increased ASCVD risk irrespective of achieved LDL-C levels. Notably, even among patients in the lowest LDL-C quartile (3.1–77.0 mg/dL), high Lp(a) conferred a 38% higher risk of cardiovascular events (HR 1.38, 95% CI 1.06–1.79). The highest risk was observed when both Lp(a) and LDL-C were elevated (HR 1.90, 95% CI 1.46–2.48), indicating that LDL-C lowering does not fully offset Lp(a)-mediated cardiovascular risk [39].

### 5.2. Impact of Lipoprotein (a) on Residual Cardiovascular Risk After Acute Coronary Syndrome

Despite robust evidence in established ASCVD, the prognostic impact of Lp(a) in the specific setting following an ACS is still uncertain, mainly due to the current absence of RCT using specific and potent Lp(a) inhibitors. The available evidence is still limited and inconsistent, with marked heterogeneity across study designs, populations, outcome definitions, and Lp(a) thresholds, complicating direct comparisons across studies. This review aims to address this gap by synthesizing the current and most recent evidence supporting Lp(a) as a major contributor to residual cardiovascular risk after ACS (Table 1) [54,55,56,57,58,59,60,61,62,63,64].

Over the past decade, several studies have demonstrated a positive association between elevated Lp(a) levels and an increased risk of adverse cardiovascular outcomes following an ACS. Yang et al. demonstrated, in a multicenter study of patients with ACS, a higher incidence of a composite of recurrent cardiovascular events (all-cause death, nonfatal MI, nonfatal stroke, and unplanned revascularization) as well as unplanned revascularization in patients with Lp(a) ≥ 30 mg/dL compared with those with Lp(a) < 30 mg/dL (*p* < 0.05), after a median follow-up of 17 months [54]. Similar findings have been reported in single-center studies using Lp(a) thresholds of ≥32 mg/dL [55], after a follow-up of more than 3 years. Long-term outcomes were evaluated by Miñana et al., who demonstrated a non-linear association between Lp(a) levels and an increased risk of very long-term reinfarction from values ≥ 50 mg/dL (*p* = 0.016), with a median follow-up of 9.9 years. However, no association was observed with all-cause mortality (*p* = 0.934) [56].

Moreover, accumulating evidence suggests that the Lp(a) threshold at which the risk of MACE increases after an ACS may be substantially lower than the conventionally defined high-risk level (≥50 mg/dL). In a cohort of 1758 patients with ACS who underwent emergency percutaneous coronary intervention (PCI), Takahashi et al. demonstrated that Lp(a) levels ≥ 15 mg/dL were associated with a significantly higher incidence of the composite endpoint of all-cause death and MI over a median follow-up of 2.2 years (HR 1.66, 95% CI 1.05–2.61, *p* = 0.03) [57]. Xue et al. concluded that Lp(a) levels > 19.1 mg/dL were associated with higher mortality in a cohort of 1359 patients over a median follow-up of 930 days (*p* < 0.001) [66]. Consistently, in a pre-specified analysis of the placebo-controlled *ODYSSEY Outcomes* trial, Steg et al. reported that Lp(a)-associated risk for MACE became evident at levels as low as 21.4 mg/dL [58]. Moreover, Lp(a) reduction with alirocumab independently predicted MACE reduction (HR 0.994, 95% CI 0.990–0.999, *p* = 0.008, per 1 mg/dL decrease) [63].

Post hoc analysis further demonstrated that, among ACS patients with elevated Lp(a), alirocumab was associated with earlier and greater reductions in both MACE and major adverse limb events (MALE) compared with patients with lower baseline Lp(a) levels. In patients with Lp(a) ≥ 30 or ≥50 mg/dL, the HR and 95% IC for MACE fell below 1 from 1.31 and 1.25 years onward, respectively, whereas no consistent benefit was observed in those with Lp(a) < 30 mg/dL, and only a delayed effect (after 3.04 years) in those with Lp(a) < 50 mg/dL. For MALE, a sustained risk reduction emerged as early as approximately 0.2 years only in patients with elevated Lp(a). This early benefit is clinically relevant, as cardiovascular risk following ACS is highest during the first year, confirming the concept that “the higher the risk, the greater the benefit” [67].

In contrast, evidence from other studies has not consistently supported an association between Lp(a) and recurrent cardiovascular events after ACS. In a post hoc nested cohort analysis of the *dal-Outcomes* trial, comparing dalcetrapib with placebo in patients with recent ACS, Lp(a) was not associated with recurrent ischemic cardiovascular events over a median follow-up of 29 months [59]. Similarly, Park et al. found no independent association between baseline Lp(a) levels and cardiovascular events in multivariable analyses of a Korean cohort with MI [60]. Gencer et al. reported that Lp(a) did not predict MACE after ACS, although higher Lp(a) levels were associated with failure to achieve LDL-C targets (<1.8 mmol/L) at one year [68]. Finally, Roth et al. observed no association between Lp(a) and either all-cause or cardiovascular mortality [69].

In specific subgroups, Lp(a) has also been reported as a predictor of adverse cardiovascular events after ACS. In patients aged ≥80 years, high Lp(a) levels (>30 mg/dL) have been associated with an increased risk of MACE, all-cause mortality, and cardiovascular mortality after long-term follow-up [61,70].

In patients with diabetes, the available evidence remains conflicting, mainly due to study limitations with potential biases and small sample size. Li et al. reported that in a cohort of patients with ST-segment elevation myocardial infarction (STEMI) undergoing PCI, diabetic patients with Lp(a) levels ≥ 30 mg/dL had a significantly higher risk of MACE during follow-up (HR 2.08, 95% CI 1.33–3.26, *p* = 0.001) compared with non-diabetic patients with elevated Lp(a) [62]. Hao et al. observed that in a cohort of type 2 diabetes patients post-ACS and PCI, Lp(a) independently predicted unplanned repeat revascularization [70]. In contrast, Silveiro et al. reported that following MI, Lp(a) was independently associated with an increased risk of the composite outcome of recurrent MI and all-cause mortality in the overall population and in non-diabetic patients, but not among those with diabetes [71].

Regarding chronic kidney disease (CKD), high Lp(a) levels have been associated with an increased risk of MACE regardless of renal function, with the risk being particularly pronounced and consistent across multiple Lp(a) thresholds in patients with CKD [72].

In patients with ACS combined with TVD, Li et al. showed that elevated Lp(a) levels remained an independent predictor of MACE (*p* < 0.05) and further described a non-linear association between Log10-transformed Lp(a) and MACE (*p* for non-linearity < 0.001), hypothesizing that very low Lp(a) levels may compromise physiological repair and anti-inflammatory processes, potentially contributing to an increased risk of adverse cardiovascular outcomes [73]. This non-linear relationship was corroborated by Wohlfahrt et al. in a cohort of 851 patients, where both individuals with Lp(a) < 2.8 mg/dL and those with Lp(a) ≥ 50 mg/dL had a higher risk of the combined endpoint of recurrent ACS and cardiovascular mortality (HR 2.6, 95% CI 1.33–5.08 and HR 2.10, 95% CI 1.00–4.39, respectively) [74].

In a systematic review and meta-analysis including 16,168 patients with ACS, elevated Lp(a) levels were independently associated with an increased risk of MACE (HR 1.26, 95% CI 1.17–1.35, *p* < 0.01) and all-cause mortality (HR 1.36, 95% CI 1.05–1.76, *p* = 0.02). Across the included studies, Lp(a) thresholds for adverse outcomes varied widely (12.5–60 mg/dL), reflecting differences in populations and measurement methods. Nevertheless, the association remained consistent, suggesting that even moderate elevations may be clinically relevant [64]. Consistent findings were reported in the systematic review by Mojahedi et al., which comprehensively evaluated the prognostic role of Lp(a) in patients with ACS undergoing PCI. Among 10 studies comprising a total of 20,896 patients, elevated Lp(a) levels—using cut-off values between 23 and 50 mg/dL—were consistently associated with both in-hospital and long-term adverse cardiovascular outcomes, independent of traditional cardiovascular risk factors, even among patients receiving statin therapy with LDL-C levels within guideline-recommended ranges [10].

Overall, recent evidence underscores the importance of Lp(a) as a predictor of adverse cardiovascular events following ACS, reinforcing the need to measure Lp(a) in routine clinical practice after an acute coronary event. Notably, Lp(a) concentrations below the high-risk threshold currently defined by clinical guidelines (15–30 mg/dL) may also be associated with an increased risk of recurrent events in the post-ACS setting. Nevertheless, the heterogeneity across studies—mainly due to the different thresholds established, the timing of Lp(a) measurement, the definitions of clinical outcomes, and the follow-up periods—limits the conclusions that can be drawn. Therefore, further large-scale, methodologically standardized studies are needed to more precisely define the prognostic value of Lp(a) after ACS and to establish clinically meaningful risk thresholds.

### 5.3. Optimal Timing for Lipoprotein(a) Assessment After Acute Coronary Syndrome

Although Lp(a) levels are generally stable over time, emerging evidence suggests that Lp(a) concentrations may transiently fluctuate during the acute phase of ACS. Therefore, the optimal timing of measurement following ACS is not yet defined. The mechanisms underlying these dynamic changes remain incompletely understood [75,76,77,78]. Ziogos et al. reported that Lp(a) levels measured six months after ACS were significantly higher than those obtained within 24 h of hospital admission, suggesting that early Lp(a) measurements may underestimate baseline cardiovascular risk [75]. On the contrary, Vavuranakis et al. observed a progressive reduction in Lp(a) concentrations one month post-ACS compared with peri- and post-infarction values, and demonstrated that this transient elevation may be mitigated by PCSK9 inhibition with evolocumab [76]. More recently, Saeki et al. described an initial decrease in Lp(a) levels within the first 12 h of ACS, followed by a rebound to baseline values at 48 h. Notably, the magnitude of this early decrease was independently associated with the occurrence of MACE over a three-year follow-up [77].

These studies suggest that Lp(a) measurements obtained during the acute phase may not accurately reflect baseline Lp(a)-associated cardiovascular risk. Therefore, confirmatory Lp(a) measure 1–4 months after the coronary event is recommended to accurately evaluate Lp(a)-related cardiovascular risk (Box 1) [5,6,7,75,76,77,78].

Box 1 **Lipoprotein(a) intra-individual variability and timing of measurement after acute coronary syndrome. Abbreviations: Lp(a): Lipoprotein(a); ACS: Acute Coronary Syndrome.**

- Significant intra-individual variability in ~20–25% of patients.
- Greatest risk reclassification at intermediate levels (30–50 mg/dL).
- Potential predictors of higher variability: baseline Lp(a), female sex, age, cardiovascular comorbidities, lipid profile, and statin use.
- Lp(a) fluctuate during ACS; acute-phase levels may not reflect baseline risk.
- Confirm Lp(a) measurement 1–4 months after ACS.

## 6. Therapeutic Strategies Targeting Lipoprotein(a)

The management of elevated Lp(a) remains a significant unmet need, with no therapies specifically targeting Lp(a) currently available. Nevertheless, the landscape is undergoing a paradigm shift with the advent of therapies that directly target the hepatic synthesis or assembly of the lipoprotein [79].

### 6.1. Current Management Strategies

Statins, the cornerstone of ASCVD prevention, are ineffective for Lp(a) lowering and may paradoxically increase levels by 10–20% via *LPA* gene upregulation [80]. However, meta-analyses confirm this does not negate their profound cardiovascular benefit, not supporting their discontinuation [81]. Similarly, bempedoic acid effectively lowers LDL-C and hsCRP but has a negligible clinical impact on Lp(a) [82].

Monoclonal antibodies against PCSK9 and small interfering RNA (siRNA) agents are the only approved pharmacologic classes known to reduce Lp(a) levels, demonstrating an approximate reduction of 20–30%, although the underlying mechanisms remain to be fully elucidated [83,84,85]. Emerging cholesteryl ester transfer protein inhibitors, such as obicetrapib, have also demonstrated mean Lp(a) reductions of approximately 36% when combined with ezetimibe [86]. However, the clinical implications of Lp(a) reduction with these agents for cardiovascular outcomes remain to be established.

Currently, the only treatment specifically approved to reduce Lp(a) levels is lipoprotein apheresis (LA), which is indicated for refractory cases with progressive cardiovascular disease despite optimal medical therapy. LA acutely removes more than 60% of circulating Lp(a) per session. Moreover, long-term longitudinal studies have demonstrated that LA is associated with a significant reduction in the rate of MACE. However, its use is limited by high cost, logistical complexity, and restricted availability. In addition, the current evidence is confined to observational studies, as randomized controlled trials are lacking due to ethical and logistical challenges [87,88,89,90].

Given the challenges in normalizing concentrations, mitigating downstream thrombotic and inflammatory risks is critical. Due to the homology between apo(a) and plasminogen, aspirin has emerged as a targeted strategy; data from the *ASPREE* and *MESA* studies suggest that aspirin significantly reduces events in primary prevention, specifically in individuals with elevated Lp(a) or high genetic risk [91,92].

### 6.2. Emerging Targeted Therapies

Novel agents leverage advanced delivery platforms, primarily N-acetylgalactosamine conjugation, to enable selective uptake by hepatocytes via the asialoglycoprotein receptor. Comprehensive details regarding the design and current status of ongoing clinical trials for the agents discussed below are provided in Table 2 [93,94,95,96,97,98,99].

The most advanced class of therapeutics involves silencing the *LPA* gene transcript. Pelacarsen, an ASO, binds to *LPA* messenger RNA (mRNA) and triggers its degradation through the nuclear enzyme RNase H1. In pivotal clinical trials, this stoichiometric mechanism resulted in approximately an 80% reduction in Lp(a) levels, with 98% of patients achieving physiological targets (≤50 mg/dL). Notably, pelacarsen reduces oxidized phospholipids, which is expected to further lower thrombotic and inflammatory risk. Moreover, ASOs have prolonged half-lives (3–4 weeks) due to their resistance to cleavage, allowing for less frequent dosing schedules [100,101]. The results from the phase 3 *HORIZON–Lp(a)* trial (NCT04023552) are awaited (anticipated February 2026); this study is designed to be the first to establish whether pharmacological decrease in Lp(a) leads to a reduction in MACE in a secondary prevention setting. The *HORIZON–Lp(a)* trial enrolled 8323 patients with established ASCVD, including prior MI, ischemic stroke, or symptomatic peripheral artery disease, and elevated Lp(a) concentrations (≥70 mg/dL). Participants were randomized, in addition to optimized lipid-lowering therapy, to receive monthly subcutaneous injections of pelacarsen 80 mg or placebo over a follow-up period of 4 to 5 years. The primary endpoint is a composite of cardiovascular death, nonfatal MI, nonfatal ischemic stroke, and urgent coronary revascularization requiring hospitalization. Enrollment has been completed, with final study completion anticipated in February 2026. The trial is expected to assess the long-term clinical impact of Lp(a) reduction with pelacarsen in patients with elevated Lp(a) and established ASCVD [93].

Building upon this approach, siRNA molecules offer a distinct mechanism characterized by catalytic efficiency. Once internalized, the antisense strand of the siRNA is incorporated into the RNA-Induced Silencing Complex (RISC), which binds to and degrades the target *LPA* mRNA. Crucially, the RISC remains active after target degradation, enabling repeated cleavage of additional mRNA transcripts. This “recycling” capacity results in potent and sustained suppression of *LPA* expression. Olpasiran exploits this mechanism to achieve reductions exceeding 95%, with phase 2 data demonstrating that 100% of patients achieved Lp(a) levels < 50 mg/dL [102,103]. Similarly, zerlasiran has demonstrated sustained efficacy (>80% reduction) over extended dosing intervals of up to 24 weeks [104].

Newer generations of siRNAs are pushing the boundaries of durability. Lepodisiran and the investigational agent SRSD216 have exhibited promising results. In phase 2 trials, lepodisiran achieved a mean time-averaged reduction of 94%, with concentrations remaining >90% below baseline for nearly one year after a single dose, supporting the feasibility of annual dosing regimens [105,106]. Likewise, preliminary data for SRSD216 indicate similar potency and duration [99].

Distinct from gene-silencing approaches, muvalaplin is a first-in-class oral small molecule that inhibits Lp(a) formation by disrupting the non-covalent interaction between apo(a) and apoB-100. By blocking this assembly at the hepatocyte surface, it reduces circulating Lp(a) levels without affecting plasminogen. The phase 2 *KRAKEN* trial validated this oral approach, demonstrating that a 240 mg daily dose reduced intact Lp(a) by 85.8%, allowing 97% of high-risk patients to reach target levels, with a safety profile comparable to placebo [107].

The ultimate frontier involves the transition from chronic suppression to a potential single-course cure. CTX320 utilizes CRISPR-Cas9 technology, delivered via lipid nanoparticles, to induce permanent disruption of the *LPA* gene in the liver. Preclinical data have demonstrated a >95% reduction in plasma Lp(a) following a single infusion, which remained stable over long-term follow-up, suggesting the possibility of permanent correction of this genetically determined risk factor [108].

In general, these targeted future therapies demonstrate unprecedented potential, offering deep (>90%), and sustained reductions in Lp(a) levels. If this biological effect also translates into a clinical benefit through hard clinical endpoint reduction in ongoing trials, these agents may redefine the standard of care.

## 7. Clinical Practice Guidelines and Consensus Recommendations

The major scientific societies concur in recognizing Lp(a) as an independent determinant of cardiovascular risk and in recommending its incorporation into clinical risk assessment. The ESC and EAS, in the 2025 update of their dyslipidemia management guidelines, recommend measuring Lp(a) at least once during adulthood, with particular relevance in young individuals with premature cardiovascular disease, familial hypercholesterolemia, or a family history of early-onset cardiovascular disease. They also note that Lp(a) levels may increase after menopause, and therefore, making repeat measurements in this setting appears justified. Guidelines further recommend cascade screening of first-degree relatives when elevated concentrations are identified. In these guidelines, Lp(a) levels ≥ 50 mg/dL are considered a cardiovascular risk modifier (Class IIa, Level of Evidence B), with the potential to reclassify risk, particularly in individuals at moderate risk or near therapeutic decision thresholds [8].

Similarly, the 2018 guidelines from the AHA and the American College of Cardiology recognize Lp(a) as a risk-enhancing factor. They recommend its measurement in individuals with a personal or family history of premature cardiovascular disease not explained by traditional risk factors, and consider levels ≥ 50 mg/dL to be clinically relevant, potentially influencing therapeutic decision-making, especially in patients at intermediate cardiovascular risk [9].

Nevertheless, the lack of robust evidence regarding the role of Lp(a) after ACS limits its incorporation into current ESC and AHA ACS guidelines. Although these guidelines strongly emphasize aggressive secondary prevention strategies—such as intensive LDL-C lowering and comprehensive risk factor control—they do not yet provide specific recommendations on Lp(a) measurement or management, instead referring to general cardiovascular prevention and dyslipidemia guidelines [109]. The forthcoming results of ongoing cardiovascular outcomes trials evaluating Lp(a)-targeted therapies [92,95] are expected to support the incorporation of specific recommendations into future ACS and secondary prevention guidelines.

## 8. Knowledge Gaps and Future Research Directions

Despite the growing evidence supporting the role of Lp(a) in residual cardiovascular risk after ACS, several key areas remain insufficiently understood and represent important priorities for future research.

First, the optimal Lp(a) threshold associated with clinically meaningful risk after ACS has yet to be clearly established. Although current guidelines identify ≥50 mg/dL as a high-risk category, emerging data suggest that lower concentrations may already confer increased risk in ACS populations. Large prospective studies using standardized measurement techniques and uniform outcome definitions are needed to define clinically actionable thresholds in the post-ACS setting.

Second, the prognostic relevance of intra-individual variability in Lp(a) levels remains unclear. While variability appears particularly relevant in patients with intermediate concentrations, it is unknown whether changes in Lp(a) levels over time are independently associated with cardiovascular outcomes or should guide clinical decision-making.

Third, the optimal timing for Lp(a) assessment after ACS requires further clarification. Available studies suggest transient fluctuations during the acute phase; however, the optimal timing for repeat measurement has yet to be clarified, with current evidence generally recommending reassessment between 1 and 3 months after the acute event.

Finally, ongoing cardiovascular outcome trials with RNA-based therapies targeting Lp(a) will be crucial to determine whether Lp(a) reduction is associated with improvements in hard cardiovascular endpoints. The results of these studies could modify the current paradigm of Lp(a), shifting its role from a cardiovascular risk biomarker to a modifiable therapeutic target in secondary prevention.

## 9. Conclusions

Residual cardiovascular risk after an ACS remains a substantial unmet clinical challenge, driven not only by LDL-C but also by other atherogenic lipoproteins, persistent inflammation, metabolic dysregulation, and prothrombotic states. Among these factors, Lp(a) has emerged as a major and independent contributor to residual cardiovascular risk.

Accumulating evidence indicates that Lp(a)-associated risk after ACS may become clinically relevant at concentrations below the conventional high-risk threshold (≥50 mg/dL), with several studies reporting increased MACE risk at levels as low as 15–30 mg/dL. Nevertheless, the heterogeneity among studies limits the ability to establish a definitive post-ACS Lp(a) threshold. Dedicated, methodologically standardized research is needed to draw firm conclusions.

Transient fluctuations may occur during the acute phase of ACS; therefore, Lp(a) measurement may optimally be reperformed 1–3 months after the index event to accurately assess baseline Lp(a)-related cardiovascular risk.

While currently available therapies have limited effects on Lp(a), novel targeted approaches—including ASO, siRNAs, oral inhibitors, and emerging gene-editing strategies—have demonstrated unprecedented, profound, and sustained reductions in Lp(a) concentrations. Ongoing outcome trials will clarify whether Lp(a) lowering translates into meaningful clinical benefit; Lp(a) may evolve from a risk marker to a modifiable therapeutic target.

## Figures and Tables

**Table 1:** Summary of studies evaluating the impact of lipoprotein(a) after acute coronary syndrome.

Study (Year)	Design	Pop.	STEMI	Mean of Lp(a) (mg/dL)	Lp(a) Threshold (mg/dL)	Median Follow-up	Outcome	Conclusion
Yang et al. (2022) [54]	Retrospective multicenter cohort	765	431 (56.3%)	13.41	≥30	1.4 y	Composite of all-cause death, nonfatal MI, nonfatal stroke, and UCR	Each 1-SD increase in Lp(a) predicted composite outcome (HR 1.29, 95% CI 1.11–1.48, *p* = 0.001), and UCR (HR 1.59, 95% CI 1.31–1.93, *p* = 0.001).
Dai et al. (2023) [55]	Prospective cohort	262	NR	21	≥32	4.6 y	Composite of CV death, nonfatal MI, and readmission for HF	Lp(a) ≥ 32 mg/dL predicted composite outcome (HR 2.84, 95% CI l 1.25–6.60, *p* = 0.01).
Miñana et al. (2024) [56]	Retrospective cohort	1223	417 (34.1%)	28.8	Q1 ≤ 8.9, Q2 9–21.6, Q3 21.6–40, Q4 40–74.6, Q5 74.6–305	9.9 y	MI	Lp(a) ≥ 50 mg/dL nonlinearly associated with MI (*p* = 0.016); not associated with long-term all-cause mortality.
Takahashi et al. (2022) [57]	Prospective cohort	1758	872 (77.1%)	15	≥15	2.2 y	Composite of all-cause death and MI	Risk of composite endpoint was higher in high Lp(a) group (≥15 mg/dL).
Steg et al. (2025) [58]	Post hoc placebo-group *ODYSSEY Outcomes*	9149	3235 (35.4%)	21.4	NR	2.8 y	Composite of CAD death, nonfatal MI, fatal/nonfatal stroke, and UA	Lp(a) > 21.4 mg/dL predicted composite endpoint.
Schwartz et al. (2018) [59]	Post hoc nested cohort analysis (dal-OUTCOMES)	4139	NR	12.3	NR	2.4 y	Composite of CAD death, nonfatal MI, UA, resuscitated CA, and fatal/nonfatal stroke	Lp(a) not associated with ischemic cardiovascular events.
Park et al. (2023) [60]	Prospective cohort	1908	695 (36.4%)	17	I < 30, II 30–49, III ≥ 50	3 y	Composite of nonfatal MI, nonfatal stroke, and CV death	Lp(a) not associated with composite endpoint.
Zhang et al. (2020) [61]	Prospective cohort	1008 (≥80y)	NR	13	≤10, 10–30, >30	2.7 y	CV death	Lp(a) > 30 mg/dL linked to CV death (HR 1.52, 95% CI 1.08–2.13, *p* = 0.016), and lower event-free survival.
Li et al. (2023) [62]	Prospective cohort	1543 (677 DM, 866 non-DM)	100%	16.9 DM, 17.3 non-DM	≥30	4 y	Composite of all-cause death, recurrence of MI, and stroke	MACE increased linearly above Lp(a) 16.9 mg/dL in DM; no effect in non-DM.
Bittner et al. (2020) [63]	Pre-specified analysis of *ODYSSEY Outcomes*	18,924	6536 (34.5%)	21.2	Q1 < 6.7, Q2 6.7–21.2, Q3 21.2–59.6, Q4 > 59.6	2.8 y	Composite of CV death, nonfatal MI, stroke, and UA	Alirocumab lowered Lp(a) by 5 mg/dL and reduced MACE (HR 0.85, 95% CI 0.78–0.93, *p* < 0.001)
Wang et al. (2024) [64]	Meta-analysis	18,168	NR	NR	From 12.5 to 60	2.9–66 m	Composite of all-cause death, stroke, non-fatal MI, and UCR	Lp(a) associated with increased MACE (HR 1.26, 95% CI 1.17–1.35, *p* < 0.001), and all-cause mortality (HR 1.36, 95% CI: 1.05–1.76, *p* = 0.02).

Abbreviations: STEMI: ST-Segment Elevation Myocardial Infarction; Lp(a): Lipoprotein(a); MI: Myocardial Infarction; UCR: Unplanned Coronary Revascularization; SD: Standard Deviation; HR: Hazard Ratio; CI: Confidence Interval; NR: Not Reported; y: Years; CV: Cardiovascular; HF: Heart Failure; Q: Quartile; CAD: Coronary Artery Disease; UA: Unstable Angina; CA: Cardiac Arrest; DM: Diabetes Mellitus.

**Table 2:** Ongoing and upcoming clinical trials investigating emerging therapies targeting lipoprotein(a). All included studies are double-blind, placebo-controlled, and phase III trials, except SRSD216 Study, which is a phase I/II study.

Molecule	NCT Number/Trial Name	Inclusion Criteria	Primary Outcomes	Estimated Completion
Pelacarsen [93]	NCT04023552*Lp(a)HORIZON*	Lp(a) ≥ 70 mg/dL Age 18–80 y ASCVD	Time to first MACE with Lp(a) ≥ 70 mg/dL or ≥90 mg/dL	Feb. 2026
Lepodisiran [94]	NCT06292013*ACCLAIM-Lp(a)*	Lp(a) ≥ 70 mg/dL Age ≥ 18 y with ASCVD or ≥55 y with high CV risk	Time to first MACE	Mar. 2029
Muvalaplin [95]	NCT07157774*MOVE-Lp(a)*	Lp(a) ≥ 70 mg/dL Age ≥ 18 y ASCVD or high CV risk	Time to first MACE	Mar. 2031
Olpasiran [96]	NCT05581303*OCEAN(a)-Outcomes*	Lp(a) ≥ 80 mg/dL Age 18–85 y ASCVD	Time to first CHD death, MI, or urgent coronary revascularization	Dec. 2026
Olpasiran [97]	NCT07136012*OCEAN(a)-PreEvent*	Lp(a) ≥ 80 mg/dL Age ≥ 50 y Multiple ASCVD risk factors and/or atherosclerosis	Time to first CHD death, MI, or urgent coronary revascularization	Oct. 2031
Pelacarsen [98]	NCT06813911 *ADD-VANTAGE*	Lp(a) ≥ 70 mg/dL Age 18–80 y ASCVD LDL-C > 70 mg/dL despite statins Run-in period of inclisiran	Change in Lp(a) levels	Dec. 2027
SRSD216 [99]	NCT07172646 *SRSD216 Study*	Age 18–70 y BMI 18–40 kg/m^2^	Incidence of treatment-emergent adverse events	Apr. 2027

Data regarding study design, inclusion criteria, and estimated completion dates were retrieved from the U.S. National Library of Medicine (ClinicalTrials.gov) database, accessed January 2026. Abbreviations: NCT: National Clinical Trial; Lp(a): Lipoprotein(a); y: Years; MI: Myocardial Infarction; MACE: Major Adverse Cardiovascular Event (cardiovascular death, nonfatal myocardial infarction, nonfatal ischemic stroke, and urgent coronary revascularization); Feb.: February; ASCVD: Atherosclerotic Cardiovascular Disease; CV: Cardiovascular; Mar.: March; CHD: Coronary Heart Disease; Dec.: December; Oct.: October; LDL-C: Low-Density Lipoprotein Cholesterol; BMI: Body Mass Index; Apr.: April.

## Data Availability

No new data were created or analyzed in this study. Data sharing is not applicable to this article.

## Abbreviations

The following abbreviations are used in this manuscript:

ACS	Acute Coronary Syndrome
MACE	Major Adverse Cardiovascular Event
LDL-C	Low-Density Lipoprotein Cholesterol
Lp(a)	Lipoprotein(a)
ASCVD	Atherosclerotic Cardiovascular Disease
RCT	Randomized Clinical Trial
PCSK9	Proprotein Convertase Subtilisin/Kexin Type 9
CAVD	Calcific Aortic Valvular Disease
ESC	European Society of Cardiology
EAS	European Atherosclerosis Society
AHA	American Heart Association
ASO	Antisense Oligonucleotide
siRNA	Small Interfering RNA
ApoB-100	Apolipoprotein B-100
Apo(a)	Apolipoprotein(a)
KIV	Kringle IV
CAD	Coronary Artery Disease
CNV	Copy Number Variation
HDL-C	High-Density Lipoprotein Cholesterol
HsCRP	High-Sensitivity C-Reactive Protein
HR	Hazard Ratio
CI	Confidence Interval
TVD	Three-Vessel Disease
MI	Myocardial Infarction
PCI	Percutaneous Coronary Intervention
MALE	Major Adverse Limb Event
STEMI	ST-Segment Elevation Myocardial Infarction
CKD	Chronic Kidney Disease
mRNA	Messenger RNA
LA	Lipoprotein Apheresis
RISC	RNA-Induced Silencing Complex
